# Perception of Speech by Individuals with Parkinson's Disease: A Review

**DOI:** 10.4061/2011/389767

**Published:** 2011-09-22

**Authors:** Lorinda C. Kwan, Tara L. Whitehill

**Affiliations:** Division of Speech and Hearing Sciences, The University of Hong Kong, Pokfulam Road, Hong Kong

## Abstract

A few clinical reports and empirical studies have suggested a possible deficit in the perception of speech in individuals with Parkinson's disease. In this paper, these studies are reviewed in an attempt to support clinical anecdotal observations by relevant empirical research findings. The combined evidence suggests a possible deficit in patients' perception of their own speech loudness. Other research studies on the perception of speech in this population were reviewed, in a broader scope of the perception of emotional prosody. These studies confirm that Parkinson's disease specifically impairs patients' perception of verbal emotions. However, explanations of the nature and causes of this perceptual deficit are still limited. Future research directions are suggested.

## 1. Introduction

Parkinson's disease is generally believed to be caused by a loss of dopaminergic cells in the substantia nigra pars compacta of the basal ganglia [1] Reduction of dopamine limits the ability of the basal ganglia to coordinate inhibitory and excitatory neural motor signals in cortical-subcortical circuits. The motor consequences of such malfunction are rigidity, tremor, and dyskinesia.

 Speech production is also affected by Parkinson's disease, resulting in hypokinetic dysarthria, which is characterized by monoloudness, monotone, and unclear articulation [2–6]. Approximately 70%–75% of individuals with Parkinson's disease exhibit speech disorder at some stage of the disease [7, 8]. However, the motor speech disorder does not necessarily correlate with the disease severity [9]. The results of medical, surgical, and deep-brain stimulation treatments of dysarthria in Parkinson's disease have been variable and generally disappointing [8, 10]. Several studies have suggested that the pathophysiology of speech disorder may be different from limb movement disorders in Parkinson's disease, including studies employing functional imaging [11, 12], demonstrating a negative correlation between disease severity and impaired speech [13], and showing nonresponsiveness towards levodopa in people with Parkinson's disease-induced oral festination [14]. However, other studies found mixed results [15, 16]. More studies are needed to explore this area [8].

Recent pathophysiological research studies have added new knowledge to the original dopamine depletion theory. Parkinson's disease is now seen as a complex neurodegenerative disease. H. Braak and E. Braak [17] suggested a sequence of pathophysiological progression of Parkinson's disease that affects first the dorsal motor nucleus of the vagus nerve and the olfactory bulbs and nucleus, then the locus coeruleus, and later on the substantia nigra pars compacta. The cortical area is affected at a later stage. Accordingly, damage to the basal ganglia, a collection of multiple neuronal systems, can result in multifaceted pathophysiological changes that cause impairments not just to motor system but also to the cognitive and neuropsychological systems [18]. 

In recent years, there has been increasing interest in nonmotor aspects of Parkinson's disease. Recent studies have identified deficits in cognitive function [19–23], neuropsychiatric status [24], and language [25–27]. There has been a parallel development in the domain of speech, as researchers have turned from a focus purely on speech production deficits to an interest in possible deficits of speech perception in this population. 

For some time, there have been anecdotal reports of a distorted perception of one's own loudness in individuals with Parkinson's disease [28]. This is manifest as an overestimation by speakers with Parkinson's disease of their own loudness (usually in the presence of reduced loudness of their speech) and/or a perception by individuals with Parkinson's disease that they are shouting or producing abnormally loud speech, when asked to produced speech of “normal” loudness, as judged by the speech-language pathologist or another nonimpaired communication partner. Another common observation is the ability of individuals with Parkinson's disease to improve their loudness (and other aspects of speech production) when prompted to do so, in a clinical or laboratory setting, but with a return to reduced loudness and poorer speech production upon leaving the clinical setting. This phenomenon has posed some challenges for research studies, as discussed later in this paper. 

The anecdotal reports of impaired perception of one's own loudness in individuals with Parkinson's disease raise a number of questions: (a) is there empirical evidence to support the anecdotal reports? (b) Is the deficit restricted to perception of loudness, or does it also manifest in other aspects of speech (such as the perception of pitch or duration)? (c) Is the deficit restricted to the individual's own speech, or is there a more general deficit, affecting the perception of others' speech? (d) What are the possible explanations for the deficit? 

In the following sections, we review the available evidence to address each of these questions. A parallel development has been a recent research interest in a possible deficit of the perception of emotion by individuals with Parkinson's disease. Although much of the research on emotion has focused on deficits in visual perception (e.g., on recognizing emotions such as angry or sad, based on photographs of faces), there has also been some attention to the perception of emotion as conveyed in speech. As the transmission of emotion through speech is conveyed using dimensions such as loudness, pitch, and duration, we also summarize recent findings from this line of research in this paper.

## 2. Evidence for a Deficit in the Perception of Loudness

One of the earliest studies of perception of loudness was conducted by Fox and Ramig [28], who asked participants with Parkinson's disease and healthy controls to self-rate their voice (loudness, shakiness, hoarseness, and monotone), speech (slurred and mumbled), and communication (understood by others, participation in conversation, and initiating conversation), using visual analogue scales. In contrast to what these researchers had observed in clinical settings, where patients frequently denied a speech deficit or claimed their speech was relatively unimpaired, the subjects with Parkinson's disease rated themselves significantly more impaired than the healthy controls on all dimensions of communication. Fox and Ramig [28] suggested possible internal and external factors that may have contributed to the discrepancy between the findings of this study and what they observed clinically. Internally, people with Parkinson's disease may be impaired in their sense of effort when they speak and may tend to deny accusations of lowered loudness, as reported by others. Externally, individuals with Parkinson's disease may rate themselves more disabled in communication because of their increasing experience of a communication partner's requests to repeat themselves. 

However, in another study, patients with Parkinson's disease who did not undergo deep-brain stimulation and patients before and after deep-brain stimulation were found to be able to rate their increasing speech impairment across time in high correlation with listener's rating of intelligibility [29]. 

A few more recent studies have used experimental procedures to investigate the perception of loudness by individuals with Parkinson's disease. Ho et al. [30] investigated individuals with Parkinson's disease who spoke softer than healthy controls. The participants with PD demonstrated inaccurate estimations of their speech loudness in two different conditions: immediate and delayed playback. The participants were asked to read aloud passages and engage in conversations at three loudness levels: soft, normal, and loud. They were then asked to estimate their own speech loudness in two conditions: (i) immediately after each reading or conversation task and (ii) after listening to an audio-recorded playback. There was no significant task difference (passage reading versus conversation) for either group, and no significant difference between the immediate and delayed conditions. However, in all conditions, there was a significant difference between the Parkinson's disease and control groups; the patients with Parkinson's disease overestimated their loudness in all conditions, relative to their actual loudness (objectively measured intensity). Ho and associates hypothesized the existence of a “decreased motor set” in people with Parkinson's disease with both hypophonia and deficient scaling in limb movements, linking motor speech control with motor limb control. They also suggested that the lack of awareness of their own reduced speech loudness by the speakers with Parkinson's disease supports the notion of cooccurrence of dysarthria and a perceptual deficit of speech. Ho and colleagues raised the question of whether impaired speech production is driven by a basic perceptual deficit or if perception is abnormal as a consequence of an impaired generation of speech mechanism that is associated with Parkinson's disease.

A further study by Ho and associates [15] suggested that individuals with Parkinson's disease who had been diagnosed with hypophonia were able to speak louder when their attention was deliberately directed to an explicit cue. Ho and associates compared 12 participants with Parkinson's disease and hypophonia and 12- age and gender-matched healthy controls on regulating their own speech loudness in reading and conversation tasks in two conditions: (1) implicit cues (background noise and immediate auditory feedback) and (2) explicit cues (instruction regarding volume level). Healthy control subjects demonstrated a Lombard effect by raising their loudness against increasing background noise and decreased their loudness in response to increased immediate auditory feedback. The participants with Parkinson's disease, on the other hand, demonstrated overall softer speech than the healthy controls were significantly less capable to increase their loudness to compete with increasing background noise and to decrease their loudness in response to immediate auditory feedback. In another further experiment with an explicit attention-driven cue (instruction with regard to volume) and implicit cues, the participants with Parkinson's disease were able to increase their loudness with respect to increased background noise and were able to decrease their loudness with increased immediate auditory feedback, similar to the capacity for loudness regulation of the healthy controls. Ho and associates suggested that people with Parkinson's disease were not aware of their softer voice and produce softer voice unless explicit cues were given. Ho and associates supported the notion that the deficiency in scaling speech loudness was similar to the deficiency in scaling limb movements in Parkinson'sdisease. The improvement in loudness regulation by raising attentional effort observed in this study suggests the involvement of executive function [31]

Dromey and Adams [32] compared the performance of adults with Parkinson's disease with that of healthy controls in three tasks: loudness estimation of warbled pure tones, loudness rating of subjects' own voices against intensity anchors, and production of sustained phonation at a range of predetermined loudness levels. There was no statistically significant difference in the perception of loudness between the subjects with Parkinson's disease and the healthy controls on any of the three tasks. The authors suggested that the lack of significant difference might have been due to the laboratory-based nature of the tasks or to the fact that the Parkinson's disease participants might have overcome a loudness perception deficit by the presence of external cues shown by the displays of the microphone amplifiers. They called for further investigations using more naturalistic communication paradigms. 

A study by Adams et al. [33] supported the notion of underestimation of speech loudness in individuals with Parkinson's disease. Participants with Parkinson's disease were asked to converse for 2-3 minutes in five noise conditions (multitalker noise varied from 50 to 70 dB SPL). The participants raised their vocal volume as background noise increased. However, their intensity levels were 3-4 dB less than the loudness increases by healthy controls under the same background noise level, representing a significant difference. 

 Two further studies have provided indirect evidence for the perception of loudness by patients with Parkinson's disease, by focusing on how patients respond to external cues. Ho et al. [34] investigated the effect of interlocutor distance on the perception and production of speech loudness by people with Parkinson's disease. Twelve patients and twelve healthy controls were first asked to estimate the loudness of a playing tape recorder that was moved away from them at various distances (randomly from 1 m to 8 m). The participants were asked to estimate the loudness of the moving tape recorder by dialing the volume knob of another tape player playing the same speech sample as the one that was moving at various distances from them. They could not see the moving tape recorder. All participants estimated that the loudness of the tape player decreased with increasing distance but the participants with Parkinson's disease estimated the loudness level to be significantly greater than that estimated the control participants for the same distance between the tape player and the listeners. The reason for such a discrepancy was not clear. In another task, the participants were asked to carry out a conversation and produce sequenced speech tasks (e.g., counting days of the week) with an experimenter. The experimenter varied his distance from the participants (1 m to 8 m, varied randomly) during the two speech tasks. In both the conversation and the sequenced speech task, the participants were audio-taped and speech loudness was measured. All participants raised their speech volumes in either conversation or sequenced speech tasks interlocutor distance increased. However, the Parkinson's disease group produced significantly softer voice than the healthy controls in both speech tasks. Furthermore, the loudness increase in conversational speech was less than the loudness increase in the sequenced speech task in the Parkinson's disease group. This phenomenon was explained by the authors as patients with Parkinson's disease had more difficulties to estimate their speech loudness when they focused more on the content of speech (as in conversation) rather than focusing only on speech loudness (as in the more predictable sequence speech). Ho et al. [34] attributed the significant reduction in loudness to apossible sensori-motor disintegration similar to the dampened movement baseline in limb motor set production found in Parkinson's disease [35]. Parkinson's disease patients may have a general impairment in sensorimotor integration which is manifested in reduced stride regulation in walking as well as in reduced speech loudness regulation [15, 34]. 

Similar to these findings, Adams et al. [36] found that ten participants with Parkinson's disease had a significant and parallel increase in loudness in conversation in two conditions: (i) presence of multitalker noise and (ii) presence of an interlocutor moving away at distances from 1 m to 6 m randomly. The participants with Parkinson's disease had significantly less increase in loudness (3-4 dB) than the 14 healthy controls in both conditions. However, in their third condition—a concurrent manual task of hand squeezing an air bulb attached to a pressure transducer—the researchers found that the participants with Parkinson's disease increased loudness significantly more than the controls. In contrast, the controls reduced speech intensity when concurrently manual task was carried out. The reason for this apparent enhancing effect of a concurrent manual task on speech loudness regulation in Parkinson's disease was not known.

In summary, several empirical studies have provided support for the clinical observation that people with Parkinson's disease lack awareness of their impaired loudness and have difficulties in appropriately adjusting loudness. However, other studies have failed to support this notion. Discrepancies in findings are probably related to the nature of the tasks involved, although differences in severity between study groups cannot be ruled out. Most studies have focused either on the perception of external speech signals by people with Parkinson's disease or this group's evaluation of their own loudness. It is still unclear whether people with Parkinson's disease have a perceptual deficit of loudness relative to their own speech only or whether the possible deficit also affects the perception of loudness in other people's speech (although anecdotal evidence would suggest the former). A few investigators have suggested possible explanations for an impairment in the perception of loudness in individuals with Parkinson's disease; these will be further reviewed in a later section. 

Compared to reports of deficits in the perception of speech loudness, there are relatively few studies of perceptual deficit affecting other specific dimensions of speech [31]. Fox and Ramig [28] found that patients with Parkinson's disease ranked loudness as one of the most impaired dimensions of their voice, speech, and communication; there have been no other systematic comparisons. However, several studies have focused on the perception of prosody of speech, which involves the perception of pitch, rate, loudness, and duration [37–39]. Most investigations of the perception of prosody by persons with Parkinson's disease have been concerned with the perception of emotional prosody. These studies are reviewed in the section below.

## 3. Perception of Emotion and Prosody

The perception of emotion by persons with Parkinson's disease has attracted a great deal of research attention in recent years. Several studies have now provided evidence of deficits in the perception of emotions [40–42]. The deficits appear to be specific to basic emotions such as anger, fear, disgust, happiness, sadness, and surprise, rather than more complex emotions such as jealousy or admiration [43, 44]. Other studies, however, were unable to provide support for a perceptual deficit for emotion [45–48]. Furthermore, while some studies have found that the deficit affecting perception of emotion manifested itself in at least two major sensory modalities, visual perception of facial expressions and auditory perception of speech prosodies [40, 49], other studies were only able to identify perceptual deficit of emotions in just one modality, for example, in speech only [50] or in facial expression only [51, 52]. However, a more recent study by Paulmann and Pell [53] suggested that participants with Parkinson's disease were less accurate to recognize emotions than healthy controls irrespective to modality (voice, facial expressions, or semantic-lexical) or combinations of the modalities.

Gray and Tickle-Degnen [54] conducted a meta-analysis of 34 studies, incorporating 1295 participants with Parkinson's disease, on emotion recognition in people with Parkinson's disease. The effect size of their analysis was 0.52 (*g* = 0.52) suggesting a disease-specific perceptual deficit of emotions in facial expression and voice in Parkinson's disease. Their analysis confirmed the presence of a deficit in the perception of emotion in speech, especially affecting the perception of negative emotions such as anger and disgust (see also [49, 50, 55]. This lends support to the earlier conclusions by Lloyd [56], Pell [38], Pell and Leonard [39], Breitenstein et al. [37, 57], Scott et al. [58], and Yip et al. [59]. Gray and Tickle-Degnen [54] further found that the deficit is not likely secondary to depression. Below, we summarize a few key studies focusing on the perception of emotion in speech. 

 Scott et al. [58] conducted one of the earliest studies to investigate a possible speech prosody perceptual deficit in people with Parkinson's disease. Participants listened to speech samples conveying different emotions and were asked to select which facial emotion (from a choice of four) best fit their perception. The participants with Parkinson's disease, who cognitively fit, were less accurate in matching the emotionally intoned sentences with the respective facial expressions when compared with healthy controls. Participants were also asked to produce sentences with an angry emotion. The participants with Parkinson's disease were less capable of producing sentences in an angry tone, as judged by the researchers, using a 3-point scale, and were less successful in marking the prosodic differences between producing questions and statements. Scott and colleagues suggested a possible relationship between deficits in the perception and production of prosody in Parkinson's disease but did not indicate the nature of the relationship. 

However, other studies found a dissociation between production and reception of emotional prosody [46, 60]. In the study by Benke et al. [60], participants with Parkinson's disease who had no cognitive impairment were able to perceive emotion prosody (anger, sad, surprise, and cheerfulness) but were impaired in the production of emotional prosody. Contrastively, participants with Parkinson's disease with a mild to moderate degree of cognitive impairment (measured by neuropsychological tests that covered areas such as memory and nonmotor visual coordination) were found to be less able to identify emotions from prosody when compared either to nondemented participants with Parkinson's disease or healthy controls. This suggests that the cognitive level rather than production deficit may be the determining factor of deficit in perception of emotion prosody in Parkinson's disease.

A replication of Scott and associates' study found that Parkinson's disease patients had no difficulty in the recognition of vocal emotions but were impaired in the production vocal emotions [46]. The authors also suggested that the cognitive decline in Parkinson's disease is associated with a failure in comprehension of emotion prosody. Both studies (Benke and associates and Caekebeke and associates) disagreed with the notion of an association between production and reception of emotion prosody suggested by Scott et al. [58]. 

Lloyd [56] investigated nine Parkinson's disease patients who had been screened not to be demented or clinically depressed, on three experimental tasks. The participants with Parkinson's disease were not significantly different from healthy controls in phonemic discrimination tasks or lexical stress discrimination tasks but were significantly poorer in identification of emotional prosodies from neutral sentences (plain statements). Lloyd suggested that the deficit in perception of prosody in Parkinson's disease cannot be explained by phonological or lexical discrimination ability.

 Pell [38] studied the discrimination and comprehension of prosodies (linguistic: declarative and interrogative; emotional: sad, happy, and neutral) in eleven Parkinson's disease patients and found that the participants were significantly poorer than healthy controls on identification of the emotional prosodies but did similarly well as the healthy controls when asked to discriminate if two given prosodies were the same or different. This study confirms an impaired perception of prosody in people with Parkinson's disease as suggested by Scott et al. [58]. Since the participants with Parkinson's disease had difficulties with perception but not discrimination, Pell suggested that the deficit in perception of prosody in Parkinson's disease does not occur in early stage of auditory processing but in task requiring higher cognitive demands, that is, involving higher cognitive processes. However this suggestion was not supported by a study using event-related brain potentials (ERPs) to measure the brain activities of participants with Parkinson's disease when they categorized perceived emotion prosody. Schröder et al. [50] used a mismatched negativity (MMN) task, with measurements at 170–220 msec and P3b, 350–550 msec, quantified by Cz and Pz electrode sites, to study the categorization of “anger” emotions amongst two other emotions (happy and neutral) in patients in two conditions: passive and active listening. The authors explained that, in passive listening, the ERP revealed an early stage of auditory processing whereas, in active listening, ERP revealed higher cognitive function in the participants. ERP generated by emotional deviants (happy/sad) during passive listening revealed diminished amplitudes of the mismatch-related negativity for sad deviants. The authors suggested that the perception deficit for emotion prosody is early in preattentive auditory processing (when patients listened in the passive listening condition) and not at a higher cortical level. They concluded that dopamine deficiency in striato-thalamo-cortical loops is the main cause of the processing shortfall of emotive prosody found in Parkinson's disease (see also the review by Schröder et al. [61]). This study provided a pathophysiological perspective, in contrast with the behavioral perspective adopted in Pell's earlier study [38].

In summary, the deficit in perception of emotion prosody in Parkinson's disease is associated with cognitive decline [38, 46, 60] but not associated with phonological or lexical discrimination [56]. However, at least according to Pell (1996) [38], the cognitive deficit found in participants with Parkinson's disease who failed to perceive emotion prosody was not similar to dementia. This may imply that it is the cognitive skill that is associated with attention or memory resource allocation required for identification of emotions from prosodies which is impaired. Working memory and executive function are responsible for allocating memory resources in situations which require competition for attention resources. According to Repovš and Baddeley (2006) [62], working memory is part of cognitive process that provides a transient storage and manipulation of the information necessary for complex cognitive tasks such as language comprehension and reasoning. Working memory is composed of four functional components: a central executive that controls information to and from three other components: visuospatial sketchpad, episodic buffer and phonological loop. The phonological loop is responsible for storage and maintenance of phonological information, the visual-spatial sketchpad is responsible for storage and maintenance of visual and spatial information, and the episodic buffer is a multidimensional centre responsible for binding information to create integrated episodes. 

Speech perception tasks involve some degree of executive control and working memory [39, 63]. In the following section, studies on the perception of emotion prosody in people with Parkinson's disease and the association of this process with working memory and executive functions will be discussed.

Breitenstein and associates [37] hypothesized that a specific cognitive impairment such as compromised working memory and executive function may impair perception of emotion prosody. They studied 11 patients with Parkinson's disease who were nondemented (assessed by Mini-Mental State Examination [64]), had normal hearing and were of moderate disease stage (all at stage II of the Hoehn & Yahr (HY) [65]). The authors used tasks such as listening span to test working memory, alternate verbal fluency set-shifting, and card sorting to test executive function of the participants. Participants were asked to identify emotions from semantically congruent and non-congruent sentences. The semantically incongruent sentences were designed to examine participants' ability to divide attention (i.e., to distribute attentional resources by selectively focusing on the emotional prosodic meaning while inhibiting the irrelevant or contradictory semantic content of the sentences). The participants with Parkinson's disease who scored low in composite scores of working memory and executive function tests were also significantly less accurate than healthy controls in the identification of prosodic emotions from semantically incongruent sentences with neutral stimuli. Breitenstein and associates explained the findings by claiming that all these cognitive processes are subserved by the same frontostriatal circuitry in the brain. However, further explanation of this claim appears to be needed. 

This study has provided accumulating evidence that the perception of emotion prosody found in nondemented and nondepressed patients with Parkinson's disease is caused by compromised central executive function and working memory. There is still some uncertainty regarding whether a specific working memory deficit in Parkinson's disease can only be revealed by tasks which require selective attention. 

Breitenstein et al. [57] modified their previous study (1998) to further investigate the cognitive processes that underlie the processing of verbal emotions. They explored the association between a deficit in perception of emotion prosody and two areas of cognition: (1) frontal executive function; (2) acoustic processing. Twenty patients with Parkinson's disease without dementia (assessed by Mini- Mental State Examination [64]) and with normal hearing were recruited and tested on their general intellectual functioning (tested by MMSE and Vocabulary or Picture Completion IQ scores), immediate memory (tested by digit spans forward or backward), and central executive frontal lobe functions (tested by verbal fluency task and Wisconsin Card Sorting Test). The participants were further grouped into mild and moderate PD, according to the absence or presence of dopamine replacement therapy (DRT). Those with DRT were characterized as the mild PD group and those with DRT as the moderate PD group. Using similar procedures to the previous study, the participants were asked to identify emotions from semantically congruent and incongruent sentences. The participants with moderate PD group performed significantly poorer than healthy controls and the mild PD group in identification of emotions from sentences with both congruent and incongruent semantic meaning. The accuracy of identification of emotion prosody in the moderate PD group in noncongruent tasks was correlated to their composite scores in central executive functioning. However, there was no significant difference in accuracy between the congruent and noncongruent tasks for the moderate PD group. Breitenstein and associates explained that the moderate PD group might have compensatory strategies to focus on prosody rather than the semantics of the sentences, leading to their similar performance accuracy. The results were compared to those of patients with right-hemisphere stroke, who scored lower in the noncongruent tasks than the congruent tasks. The stroke patients appeared to lack the compensatory strategies of the patients with Parkinson's disease.

Finally, there was no significant difference in accuracy of emotion identification between participants in the mild PD group and healthy controls. Only patients in the moderate PD group demonstrated significantly poorer performance than the healthy controls (as well at the mild PD participants). The presence of dopamine administration in the moderate PD group could not be ruled out as a possible cofounding variable.

 In the second part of this study (Breitenstein et al. [57]), the researchers systematically manipulated two major acoustic variables that play a major role in determining emotion prosodies: speech rate and fundamental frequency (F0 variability). The participants listened to sentences spoken in German (which none of them had knowledge of) that were stepwise manipulated in rate (such that rate increased by 110–150% and decreased by 90–50%) and in fundamental frequency (increasing by 110–150% and decreasing by 90–50%). The participants rated certainty of a perceived emotion on a 5-point scale. All participants were more accurate in identification of emotions when pitch rather than rate was manipulated. However, the healthy controls were significantly better than the patients with Parkinson's disease in identification of emotions when either pitch or rate was manipulated. This result indicated that all participants relied on pitch or rate to decode vocal emotions but the Parkinson's disease patients became less successful than the healthy controls to comprehend emotions when the pitch or rate was altered. The healthy controls were also found to benefit from slower rate to decode emotions in prosodies whereas the Parkinson's disease patients were confused by slower speech rate. The authors [57], in addition to working memory and central executive function, suggested that at least one other cognitive process, acoustic tone duration processing-accounted for the perceptual deficit in people with Parkinson's disease. 

Pell and Leonard [39] also offered compelling evidence for the impact of working memory on the perception of emotional prosodies. They studied 21 nondemented Parkinson's disease patients who were at relative early stage of the disease using discrimination, identification, and intensity rating tasks. Pell and Leonard stated that the tasks of comprehension of prosody were dependent on the functioning of working memory and executive function of participants. In the perception of emotion prosody, the participants have to hold auditory information in memory and simultaneously process, generate a response, visually attend to verbal labels or other response prompts and shift mental sets across tasks that required different types of processing and response to similar auditory materials. In this study standardized neuropsychological tests that test working memory, attention, and executive function were administered. These tests included Forward Digit span (measuring verbal working memory span ) [66], the Trail-Making Test, the Wisconsin Card Sorting Test (WCST), and attention subtest of the Mattis Dementia Rating Scale. Pell and Leonard explained the necessity of using standardized neuropsychological tests because they wished to dissociate the performance in perception of emotion prosody of the participants from more basic cognitive domains. The participants were asked to discriminate emotions (same/different), identify emotions, and rate the success of intended emotions from emotionally intoned nonsense sentences spoken by five actors. The participants with Parkinson's disease recognized emotions significantly less accurately in all three tasks when compared with healthy controls. There was no significant difference in a depression index between the two groups, indicating that depression could not account for the impairment of perception of vocal emotions found in the participants with Parkinson's disease. They concluded that the perception deficit is found in patients who were nondemented. The participants with Parkinson's disease demonstrated significant association between capacities to discriminate and to identify prosody with auditory processing/working memory capabilities (both *rs* = .51, *Ps* < .05). The results supported the same notion suggested by Breitenstein and associates [37, 57] that a specific compromised working memory and executive function is more likely the cause of impaired perception of emotion prosody.

Paulmann and Pell [53] tested the hypothesis of channel availability in perception of emotion prosody in people with Parkinson's disease. The participants with Parkinson's disease in this study were tested to be cognitively high functioning as they performed well on several traditional executive function and working memory tests (e.g., Tower of London, Trail-Making Test, and verbal fluency). The authors conducted a series of perceptual tests with eleven patients with Parkinson's disease using different combination of modalities: visual, prosody, and linguistic. Participants were asked to perceive emotions conveyed in three conditions/channels: unimodal (face, prosody, or lexical), bimodal (face + prosody, prosody + lexical, or face + lexical), and trimodal (face + prosody + lexical). Both groups performed better when more channels were available. However, the Parkinson's disease patients were significantly poorer in all three conditions (uni-, bi-, and trimodal) compared with the healthy controls. The authors suggested that Parkinson's disease patients were less able to perceive emotions irrespective to modalities (voice, facial expression, and semantics-lexical) or any combination of these modalities. As the participants were tested to be well functioning in their working memory and executive function but still demonstrated a deficit in perception of emotion prosody in all three modalities, Paulman and Pell suggested other possible factors that may impinge on the perception of emotion prosody in people with Parkinson's disease who are not demented or compromised in working memory. 

The literature has been nonconclusive regarding changes in the perception of emotion prosody resulting from various pharmacological or surgical treatments. It has been suggested that the perception may be enhanced by dopamine level [67] although no empirical study has been done. Also, there was no significant difference in rating the valence (decision of whether an emotion is positive or negative) of emotion prosody between the patients with Parkinson's disease who did not undergo deep-brain stimulation and patients who had deep-brain stimulation [68].

In summary, the perception of emotions in human speech has been found to be impaired in Parkinson's disease in many studies. Combined evidence from these research studies has suggested that neither depression nor dementia accounts for the decreased perceptual accuracy of emotions in speech; impaired working memory does appear to be implicated. These studies have defined “working memory” with relevance to the widely accepted four-component working memory model proposed by Repovš and Baddeley (2006) [62]. However, interest has been mainly on testing working memory as a whole, with emphasis on the supervisory component executive function. The other components of working memory, especially the phonological loop, which is crucial to language comprehension, have not been reported as much in studies on perception of emotion prosody in people with Parkinson's disease.

In the studies that associate working memory deficit and perceptual deficit of emotion prosody in people with Parkinson's disease, working memory, and its component executive function were measured by similar (e.g., Listening Span measuring working memory and Wisconsin Card Sorting Test measuring executive function were both employed in Breitenstein et al. 2001 [57] and Pell and Leonard, 2003 [39] to test central executive function) or different neuropsychological tests (e.g., Trail making test was used by Pell and Leonard's study in 2003 [39], to test central executive function) or different neuropsychological tests (e.g., Trail making test was used in Pell and Leonard's study in 2003 [39] but not in the study by Breitenstein and associates in 2001 [57]. Therefore, methodology variance is apparent in these studies concluding the association between the deficit in perception of emotion prosody in people with Parkinson's disease. 

There were also a few studies which identified a specific cognitive deficit in people with Parkinson's disease but without dementia. This specific cognitive deficit was termed differently in various studies: “limitation in supervisory attentional system” [69], “central executive dysfunction” [70–72], or “reduced working memory capacity associated with deficits in strategic memory” [73–76] (for review, please see Breitenstein et al., 2001) [37]. It is unclear how these different specific apparent cognitive deficits impinge on the perception of emotion prosody in people with Parkinson's disease.

More studies are still needed to understand the exact nature of the mechanism underlying the perceptual deficit in decoding emotions in voice.

## 4. Explanations for Perceptual Speech Deficit in Parkinson's Disease

In the sections above, we reviewed the available evidence for the presence of a deficit in the perception of loudness and other speech dimensions (such as pitch and duration) in individuals with Parkinson's disease. In this section, we review explanations that have been offered for such a deficit. 

Fox and Ramig [28] (see also [77]) characterized the observed deficit in the self-perception of speech loudness as a deficit of “ calibration” as a result of a discrepancy between sense of effort and the produced vocal loudness. Lee Silverman Voice Therapy (LSVT) [78] focuses not only on increasing loudness but also on “recalibration” of patients' ability to accurately judge their own speech loudness. Participants who have received LSVT have been shown not only to increase their loudness, but also to generalize increased loudness outside of the clinical setting and to maintain the changes over time [77]. However, there is no direct evidence that LSVT leads to a more accurate perception by patients of their own loudness. 

A few researchers have suggested an association between production deficits and perceptual deficits in speech [30, 34, 58]. However, dissociations between production and comprehension of emotion prosody have also been reported [46, 60]. Furthermore, there has been little expansion of this idea (e.g., regarding whether the production deficit may lead to the perception deficit or vice versa). This hypothesis appears to be in need of further exploration. 

As reported above, several studies have found people with Parkinson's disease are less accurate not only in the perception of emotions conveyed by speech than healthy controls but also in the perception of emotions conveyed in facial expressions [40, 42, 59, 60, 79]. This suggests a possible general deficit in the perception of emotions in Parkinson's disease. It is not known whether this reflects a possible broader deficit in perception.

Accumulating evidence suggests that the deficit in perception of emotions in speech in individuals with Parkinson's disease might be a result of general cognitive impairment. Benke et al. [60] and Breitenstein and associates [57] found that only patients at moderate stage of Parkinson's disease demonstrated such a deficit in perception [37, 46, 60]. Their studies implied that the perception deficit is a general cognitive impairment. In addition, Caekebeke et al. [46] found a dissociation between comprehension and production of emotion prosody in nondemented patients with Parkinson's disease, implying that the weakness in perception of emotion prosody is an indication of a higher cognitive impairment and is independent of disease duration, degree of motor dysfunction, and presence of dysarthria [50].

However, other studies indicated that specific cognitive deficits such as compromised working memory, executive function, or acoustic processing impairment may be the primary cause of impairment of the perception of emotion prosody in Parkinson's disease [37–39, 57, 60]. Gray and Tickle-Degnen [54] concluded from a meta-analysis of 34 research studies that compromised working memory is a possible contributing factor to the perceptual deficit of emotion prosody in Parkinson's disease. Pastor and associates [80] as well as Gräber and associates [81] suggested a cognitive deficit in an “internal pacing mechanism” in Parkinson's disease patients that prevents them from accurate comparison of measured duration with stored reference memory traces. Kotz et al. [82] suggested a model of sensory predictability in auditory language comprehension. In their model, the basal ganglia is a crucial nonmotor part of the medial pre-supplementary-motor-area-basal-ganglia (pre-SMA-BG) circuit to enhance language comprehension by extracting external temporal cues (e.g. prosody) from speech. Without forming such predictability cues, language comprehension will be consequentially unsuccessful.

Neurophysiological studies have presented additional evidence supporting a deficit in perception of emotional prosodies in Parkinson's disease. For example, the ERP study reviewed above [50] suggested that perception deficit in vocal emotions in Parkinson's disease might occur during early preattentive process (as in passive listening) rather than higher cognitive level, a suggestion that is contrastive to behavioral findings proposed by Lloyd [56] and Pell [38].

## 5. Conclusion and Directions for Future Studies

There has been a long-standing clinical recognition of an impairment in the ability of individuals with Parkinson's disease to accurately perceive their own loudness. However, until recently, there has been little empirical evidence to support this observation. In this paper, we have reviewed the available evidence on this issue. A few studies have provided empirical support for a deficit in the perception of loudness of one's own speech [30, 34]. However, there have also been conflicting reports, which have not been able to show evidence to support the clinical observation [28, 32]. The discrepancy in results may be due to differences in methodology. In particular, there appears to be a significant difference in performance of the individuals with Parkinson's disease in “lab-based” tasks when compared with more naturalistic communication situations [32]. 

Additional evidence to advance our understanding of a possible deficit in the perception of speech has come from a number of recent studies that have investigated the perception of emotion by individuals with Parkinson's disease. Again, several of these studies have provided empirical evidence to support the notion of a deficit in the perception of speech, particularly in the dimensions of speech critical for conveying emotion [37–39, 56, 57].

Returning to the questions posed at the beginning of the paper allows us another perspective to determine the current state of knowledge in this field. (a) *Is there empirical evidence to support the anecdotal reports?* There is some evidence to support the concept, as noted above. However, additional research in this area would provide further support, and careful consideration of methodological approaches may permit resolution of some current discrepancies in findings. (b) *Is the deficit restricted to perception of loudness, or does it also manifest in other aspects of speech (such as the perception of pitch or duration)?* Evidence from recent research in prosody and emotion suggests that there is impairment in the perception of other aspects of speech. There have been few studies that have systematically compared perceptual deficits in loudness to other speech dimensions. (c) *Is the deficit restricted to the individual's own speech, or is there a more general deficit, affecting the perception of others' speech?* This question has not been directly addressed and appears critical for further investigation. (d) *What are the possible explanations for the deficit?* Several theories have been suggested, and there seems to be quite strong evidence to support the involvement of working memory. Physiologically based hypotheses to explain the perceptual deficit in speech shown by individuals with Parkinson's disease are limited, to date. 
